# Advances and Prospects in Tissue-Engineered Meniscal Scaffolds for Meniscus Regeneration

**DOI:** 10.1155/2015/517520

**Published:** 2015-06-25

**Authors:** Weimin Guo, Shuyun Liu, Yun Zhu, Changlong Yu, Shibi Lu, Mei Yuan, Yue Gao, Jingxiang Huang, Zhiguo Yuan, Jiang Peng, Aiyuan Wang, Yu Wang, Jifeng Chen, Li Zhang, Xiang Sui, Wenjing Xu, Quanyi Guo

**Affiliations:** Institute of Orthopaedics, Chinese PLA General Hospital, 28 Fuxing Road, Haidian District, Beijing 100853, China

## Abstract

The meniscus plays a crucial role in maintaining knee joint homoeostasis. Meniscal lesions are relatively common in the knee joint and are typically categorized into various types. However, it is difficult for inner avascular meniscal lesions to self-heal. Untreated meniscal lesions lead to meniscal extrusions in the long-term and gradually trigger the development of knee osteoarthritis (OA). The relationship between meniscal lesions and knee OA is complex. Partial meniscectomy, which is the primary method to treat a meniscal injury, only relieves short-term pain; however, it does not prevent the development of knee OA. Similarly, other current therapeutic strategies have intrinsic limitations in clinical practice. Tissue engineering technology will probably address this challenge by reconstructing a meniscus possessing an integrated configuration with competent biomechanical capacity. This review describes normal structure and biomechanical characteristics of the meniscus, discusses the relationship between meniscal lesions and knee OA, and summarizes the classifications and corresponding treatment strategies for meniscal lesions to understand meniscal regeneration from physiological and pathological perspectives. Last, we present current advances in meniscal scaffolds and provide a number of prospects that will potentially benefit the development of meniscal regeneration methods.

## 1. Introduction

The meniscus is one of the most commonly damaged areas of the knee joint, which was once considered a “functionless remnant of leg muscle origin” [1]. The mean incidence of meniscal injury in the United States is 66/100,000 [2, 3]. Completely removing the meniscus was the major treatment for meniscal injuries in 1889, and this treatment prevailed for nearly 80 years [4]. However, a number of follow-up radiographic studies from the late 1960s to the 1980s reported a high frequency of knee osteoarthritis (OA) after total removal of the meniscus [5–7]. Clinical follow-up results also showed knee OA in all patients 14 years after partial meniscectomy [8, 9], which is the current primary method to treat meniscal injuries.

Menisci play a crucial role maintaining knee joint function, including transmitting load, absorbing shock, stabilizing the knee joint, and providing nutrition to the joint [10–14]. It is important to ensure that meniscal integrity maintains knee joint homeostasis from a surgical treatment strategy perspective [15–19]. Tissue engineering brings new hope to restore an intact meniscus with competent function. This review summarizes meniscal structure and biomechanical properties, the relationship between meniscal lesions and the development of knee OA, lesion classifications, and therapeutic strategies from physiological and pathological perspectives. We focus on advances in tissue-engineered meniscal scaffolds and provide some regenerative strategies that may potentially benefit the development of meniscal regenerative approaches in the future.

## 2. Meniscal Structure and Biomechanical Properties

### 2.1. Meniscal Anatomy

**Figure 1:**
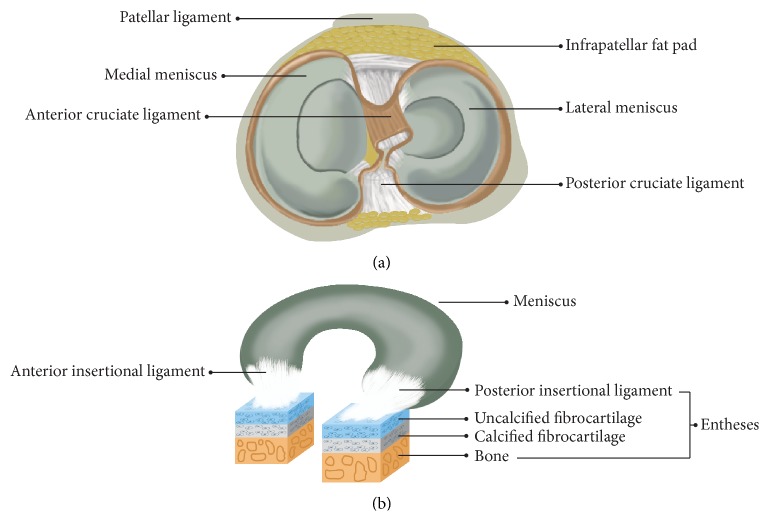
Top view of the anatomical meniscus (a): lateral meniscus is “O” shaped, whereas the medial meniscus has a “C” appearance. The meniscal functional unit (b), including the corresponding anterior and posterior ligaments and entheses. Entheses typically contain ligaments, uncalcified fibrocartilage, calcified fibrocartilage, and bone.

Menisci are a pair of crescent-shaped fibrocartilages located at the corresponding femoral condyles and tibial plateau, respectively (Figure 1(a)) [20]. The lateral meniscus covers nearly 80% of the tibial plateau area, whereas the medial meniscus only covers ~60% [21]. The geometry of the meniscus adapts well to the corresponding shape of the femoral condyle and tibial plateau. The anterior and posterior insertional ligaments play a critical role in attaching the menisci firmly, and they fix the meniscus to the tibial plateau well [22]. Blood vessels and nerves from the surrounding joint capsule and synovial tissues merely penetrate 10–25% of the outside of the adult meniscus. Therefore, the meniscus can be typically classified into three parts according to vascular and nervous distribution: the outer vascular/neural area (red-red zone), the inner entirely avascular/aneural area (white-white zone), and the junctional area (red-white zone) between the former two regions. The white-white zone does not self-heal well when damaged [23].

### 2.2. Meniscal Composition and Cell Characteristics

**Figure 2:**
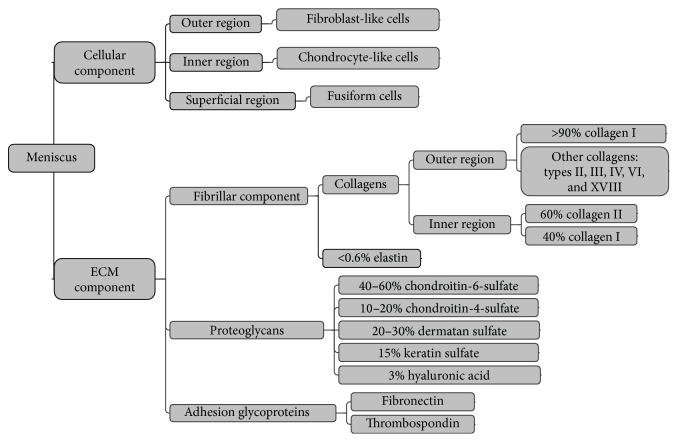
The complex composition of the meniscal cellular and meniscal extracellular matrix (ECM) components. The outer region is the outer one-third of the meniscus; the inner region is the inner two-thirds of the meniscus; the superficial region is the surface of the meniscus.

The meniscus has a highly heterogeneous extracellular matrix (ECM) and cell distribution [24–26]. Meniscal ECM components are more complex than those of articular cartilage. Cartilage has a homogeneous ECM composition, mainly comprised of water (70–80%), collagen (50–75%), and glycosaminoglycans (GAGs) (15–30%) [27]. The distribution of the meniscal ECM is categorized by region. Collagen type I accounts for >80% of the composition in the red-red region by dry weight, and the remaining content comprises < 1%, including collagen types II, III, IV, VI, and XVIII [14]. Total collagen content is 70% of the dry weight in the white-white region, whereas collagen types II and I account for 60% and 40%, respectively. The specific distribution of meniscal ECM components is shown in Figure 2.

Meniscal cell populations are classified into three types according to the different regions and cell morphology (Figure 2) [28]. The outer one-third of the meniscal area is comprised of fibroblast-like cells, which demonstrate elongated morphology. The inner two-thirds of the meniscal region mainly contain fibrochondrocytes, which are predominantly oval to round in appearance. Fusiform cells are aligned parallel to the meniscal surface in the superficial zone.

### 2.3. Meniscal Biomechanical Properties

**Figure 3:**
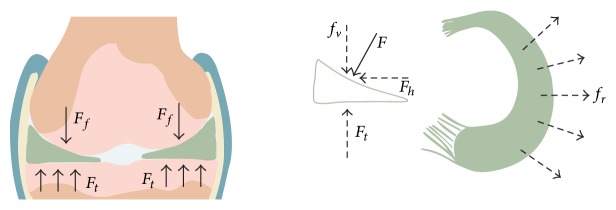
Schematic diagram of the meniscus force-bearing mechanism. Meniscal configuration adapts well to the corresponding shape of the femoral condyles and the tibial plateau in the knee joint. The axial load force (*F*) perpendicular to the meniscus surface and horizontal force (*f*_*r*_) are created by compressing the femur (*F*_*f*_). *F* rebounds due to the tibial upgrade force (*F*_*t*_), whereas the *f*_*r*_ leads to meniscal extrusion radially, which is countered by the pulling force from the anterior and posterior insertional ligaments. Consequently, tensile hoop stress is created along the circumferential directions during axial compression.

The anatomic geometry of the meniscus is closely associated with its biomechanical properties. The meniscal configuration adapts to the corresponding shapes of the femoral condyles and tibial plateau, which provide a considerable increase in contact area in the knee joint [29, 30]. Tensile hoop stress is created around the circumference when the knee bears an axial load, and this stress tries to extrude the meniscus out of the knee joint (Figure 3). However, firm attachment at the anterior and posterior insertional ligaments helps prevent extrusion of the meniscus [31, 32]. Thus, intact menisci occupy the corresponding contact area (60%) between the femoral condyles and the tibial plateau cartilage, which significantly reduces stress and protects the tibial cartilage. In contrast, if the anterior or posterior insertional ligaments or peripheral circumferential collagen fibers rupture [33], the load transmission mechanism changes, which damages the tibial cartilage.

### 2.4. Meniscal Lesions and Development of Knee OA

**Figure 4:**
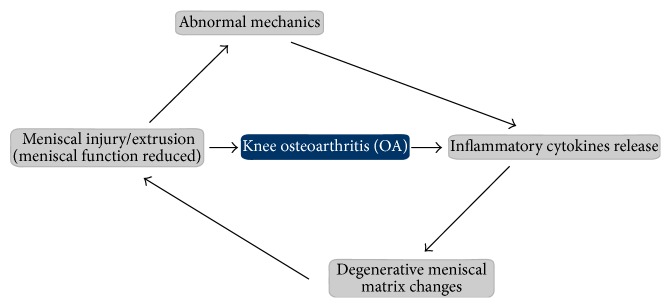
The interaction between meniscal injury and knee osteoarthritis (OA). Knee mechanics become abnormal when a meniscus is injured, which leads to increasing stress across adjacent cartilage and subchondral bone. This stress triggers release of inflammatory cytokines, which further impair the meniscal extracellular matrix (ECM) and accelerate the vicious cycle of knee OA. OA of the knee joint also causes release of inflammatory cytokines, repeating the vicious cycle.

Meniscal lesions are closely associated with the development of knee OA, and their relationship is complex [34]. Meniscal lesions can ultimately lead to knee OA, and knee OA also induces meniscal tears; therefore, normally configured menisci are rarely observed in patients with knee OA [35, 36]. An injured meniscus triggers the synovium to release various inflammatory cytokines, which induce degenerative changes in the meniscal matrix. These degenerative changes derived from early stage tears can gradually develop into meniscal extrusions that increase the stress on tibial cartilage and further aggravate the injury [24]. In addition, inflammatory cytokines released into OA joints simultaneously act on the meniscus and cartilage, as they have similar ECM components. Therefore, knee OA induced by other diseases is harmful to the meniscus and triggers similar pathological changes [25, 26, 37, 38]. Hence, a natural vicious cycle forms between meniscal lesions and the development of knee OA (Figure 4).

### 2.5. Classification of Meniscal Lesions and Therapeutic Strategies

**Figure 5:**
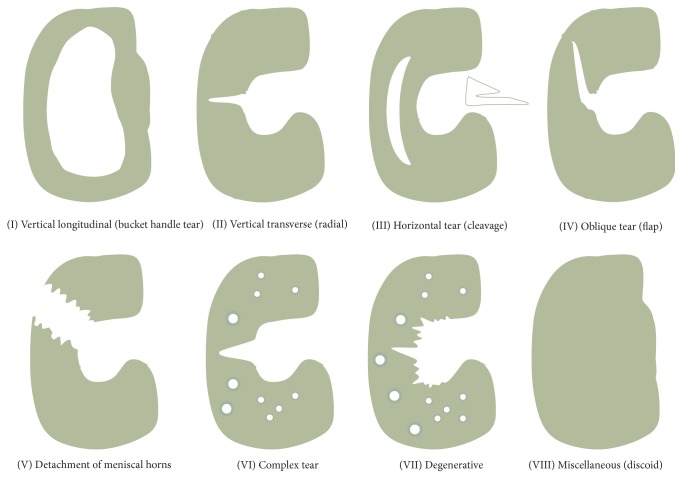
Schematic diagram of the eight different types of meniscal lesions according to Casscells classification.

Damage to the meniscus is very common in the knee joint. Meniscal lesions are typically categorized into distinct age groups. Meniscal injuries in younger patients (<40 years) are usually caused by trauma or congenital meniscal diseases, whereas those in older patients (>40 years) tend to be associated with degenerative tears [39]. In general, all meniscal lesions can be comprehensively classified into eight different types according to Casscells classification (Figure 5) [40]. However, meniscal injuries can simply be classified clinically into peripheral meniscal lesions and avascular meniscal lesions.

Orthopedic surgeons commonly perform a partial meniscectomy in cases of unrepairable or degenerative meniscal injuries [41]. However, this treatment strategy does not prevent the development of knee OA. A partial meniscectomy may decrease the contact area between the femoral condyle and tibial platform [35, 36]. Therefore, meniscal repair and reconstruction techniques have received much attention [42]. Younger patients with repairable injuries, such as longitudinal lesions or injuries in the vascular zone, are generally better candidates for meniscal repair. The types of repair procedures are inside-out, outside-in, all-inside, and repair enhancement. In contrast, an increasing number of reconstruction strategies have been developed to restore meniscal function, including meniscal allografts, small intestinal submucosa (SIS) implants, and autogenous tendon grafts. Milachowski and Wirth performed the first free meniscal allograft transplantation in 1984 [43]. Meniscal allograft transplantation enhances knee function and reduces pain significantly in relatively young patients after a short follow-up [44, 45]. However, whether meniscal allografts provide long-term protective benefits to the cartilage remains debatable, as meniscal allograft transplantations can increase the risk of disease transmission, decrease material properties, and the allografts can shrink [46]. SIS and autogenous tendon grafts have not obtained satisfactory results [47, 48].

## 3. Advances in Meniscal Scaffolds

### 3.1. The Bioabsorbable Synthetic Polymer Scaffold (Table 1)

Bioabsorbable synthetic polymers, such as polyurethane (PU), polyglycolic acid (PGA), polylactic acid, and poly (*ε*-caprolactone) (PCL), are widely used and have played a key role creating meniscal scaffolds [49]. These polymers provide several advantages, such as versatility, satisfactory biomechanical properties, and access to a nearly endless supply. However, some disadvantages of synthetic polymers include their hydrophobic properties, lack of bioactivity, and production of aseptic inflammation or an immune response.

Koller et al. attempted to enhance the bioactivity of synthetic scaffolds by adding polyethylene terephthalate (PET) to hyaluronic acid/PCL scaffolds [62]. Their results demonstrated that scaffolds with PET express more type II collagen mRNA and secrete more GAGs than those without PET. It is well known that native meniscal collagen fibers align circumferentially [11]. Baker and Mauck developed aligned (AL) scaffolds by electrospinning [63]. Cells in the AL group displayed an AL morphology, whereas those in the nonaligned (NA) group took on a polygonal shape. The biomechanical properties increased in the AL group, as compared to those of the NA scaffolds. Thus, AL scaffolds directed cell growth and enhanced biomechanical capacity. Similarly, Fisher et al. used a modified electrospinning approach to produce circumferentially AL scaffolds [64], and the results showed that seeding juvenile bovine mesenchymal stem cells (MSCs) in the scaffolds resulted in circumferential cellular alignment, similar to that seen in the native meniscus.

Koller et al. used PGA reinforced by bonding with PLGA (75 : 25) to fabricate a meniscus-like scaffold [62]. Allogenic meniscal cells were seeded into the scaffolds* in vitro* for 1 week to replace the medial meniscus in rabbits. The staining results showed that the regenerated neomenisci were similar to the native meniscus; however, the neomenisci did not prevent the tibial articular cartilage from degenerating, and cartilage degeneration was less severe in the cell-seeded group than that in the nonseeded group. Chiari et al. used hyaluronic acid and PCL in scaffolds to repair total and partial meniscal defects in sheep [65]. These scaffolds were not seeded with cells before implantation. The implant retained its morphology and remained in position for 6 weeks. The histological results showed that the implants and native menisci were integrated, and a large number of blood vessels formed to firmly bond the capsule, which was covered by synovial-like tissue. However, body weight led to extrusion and unavoidable degeneration of the cartilage in both the total and partial transplantation groups but cartilage degeneration was slightly less than that in the empty control group.

The novel biodegradable Actifit implant (Orteq Sports Medicine, London, UK) is an acellular meniscal scaffold composed of PU (20%) and PCL (80%) [66]. The interconnected pore structure enhances vessel in-growth and regeneration of the meniscus from the meniscal wall. A study using Actifit during partial meniscectomy repair in 13 skeletally mature sheep revealed regenerated tissue penetration 6–12 months after surgery [58]. Actifit has also been applied clinically to treat partial meniscal lesions. As results, dynamic contrast-enhanced magnetic resonance imaging (DCE-MRI) showed successful tissue growth into the scaffold after 3 months in 35 of 43 (81.4%) patients [67]. In contrast, 43 of 44 (97.7%) patients revealed integration with the native meniscus 12 months after surgery, and the histological results showed continuous tissue regeneration. Baynat et al. demonstrated that normal chondrocytes and fibrochondrocytes of 18 patients penetrated into the substitute 1 year post-Actifit implantation [61]. All patients were restored to their daily activities, and nine returned to their previous sporting activity level 2 years after surgery. Moreover, MRI showed no damage to the substitute or degeneration of adjacent cartilage.

## 4. Absorbable Scaffolds Derived from Biological Components (Table 2)

Absorbable scaffolds derived from biological components are very promising. They can be classified as ECM-related scaffolds and biological scaffolds.

Stone et al. reported on copolymeric collagen-based scaffolds derived from bovine Achilles tendon to repair subtotal meniscectomy in dogs without seeding cells [79]. The implanted group showed substantial meniscus-like regeneration in 15 of 24 (63%) joints, compared to 3 of 12 (25%) regenerated menisci in nonimplanted control joints. In contrast, joint gross appearance scores and India ink test scores demonstrated no significant differences. Based on these results, the collagen meniscus implant (CMI) was applied without seeding cells in a multicenter clinical trial [80]. CMIs were implanted in patients with chronic and acute meniscal injuries and compared with a partial meniscectomy group. Biopsies 1 year after implantation showed that some meniscal-like tissues had regenerated and integrated well with the host meniscal rim in the chronic group. These patients regained significantly more mobility and required fewer reoperations than those in the control group. The authors concluded that the improved clinical outcomes could be a result of regenerated meniscal-like tissues. However, CMIs did not improve the clinical outcomes of patients with acute meniscal injuries.

Monllau et al. reported the clinical outcomes of implanting CMIs after a minimum 10-year follow-up [74]. Twenty-five of their patients with a CMI substitute reported remarkable pain relief and functional improvement without any degenerative knee joint diseases in most cases. However, this was a nonrandomized trial and lacked a control group, which restricts the credibility of the results. Zaffagnini et al. conducted a cohort study during a minimum 10-year follow-up [75] and obtained similar results to the former case report. Long-term randomized controlled trials on larger populations must be carried to confirm the benefits of CMI substitution.

Tan et al. found that dedifferentiation of rat or human meniscal fibrochondrocytes can be reversed using chondroitin-6-sulfate- (C6S-) coated rather than collagen I/II surfaces during expansion of the monolayer [72, 81]. They demonstrated upregulation of collagen II and aggrecan gene expression, as well as proteoglycan production. Those authors fabricated a C6S scaffold and explored the three-dimensional (3D) conditions and oxygen tension effect on a cell-C6S scaffold construct. The results showed that the 3D cultures under hypoxic conditions strengthen fibrochondrocyte redifferentiation capacity.

Silks are a group of fibrous proteins [82] widely used in tissue engineering for chondrogenesis, osteogenesis, ligament engineering, and other aspects. Silks possess superior biomechanical capabilities, versatile processability, and good biocompatibility and controlled degradability [83]. Mandal et al. used silk fibroin from* Bombyx mori* silkworm cocoons to recapitulate a multilayered, multiporous scaffold that mimicked native meniscal architecture and morphology [76]. Human primary fibroblasts were seeded into the outside of the scaffold, and human primary chondrocytes were seeded into the inside to duplicate normal meniscal cell distributions. The results showed that the constructs increased cellularity and ECM content under chondrogenic culture conditions. In addition, the compressive modulus and tensile modulus increased with time; however, they remained inferior to those of the native meniscus. Shortly thereafter, the same authors used human bone marrow stem cells to construct tissue-engineered menisci with this multilayered scaffold and obtained similar results [77].

Bacterial cellulose (BC) is a polysaccharide synthesized by the* Gluconacetobacter xylinus* bacterium [78]. BC has numerous advantages in tissue engineering, such as superior biomechanical capacity, high hygroscopicity and crystallinity, and good biocompatibility. BC has been applied to blood, vessel, cartilage, and bone tissue engineering, as well as to treat burns [84]. Bodin et al. compared the biomechanical properties of BC gel to collagen material and a pig meniscus [85]. The compression modulus of the BC gel at 10% strain (1.8 kPa) was five times better than that of the collagen meniscal implant (0.23 kPa); however, it was inferior to the native pig meniscus (21 kPa). In another study, Martínez et al. fabricated a microchanneled BC scaffold seeded with 3T6 mouse fibroblasts and compared dynamic compression to that of a static culture [78]. The results showed that the microchannel structure directed the growth of 3T6 fibroblasts and secreted AL collagen fibers. Similarly, dynamic stimulation improved collagen production.

## 5. Hydrogel Scaffolds

Hydrogels have been used as meniscal scaffolds due to their noncytotoxic and insoluble features. Hydrogels are made of poly N-isopropyl acrylamide or alginate [14]. These materials can absorb large quantities of water (>90%), which determines their physical properties but they also exhibit great versatility, which can be beneficial for evenly mixing seed cells, loading growth factors, or creating appropriate morphology [86]. However, hydrogel scaffolds have poor tensile capacity and bioactivity.

Polyvinyl alcohol-hydrogel (PVA-H) has excellent viscoelastic properties and biocompatibility. Kobayashi et al. developed PVA-H artificial menisci to replace defective menisci. Mechanical tests confirmed that the PVA-H artificial meniscus has similar mechanical properties to those of the native meniscus [87]. A PVA-H artificial meniscus implantation group revealed normal articular cartilage conditions 1, 1.5, and 2 years after surgery in rabbit studies [88–90], and no wear or disruption of the PVA-H artificial menisci was observed. However, knee OA was detected after 1 year and continued to progress in the meniscectomy group. These results suggest that PVA-H artificial menisci are as competent as the native meniscus and have potential future clinical applications.

Grogan et al. constructed a 3D methacrylated gelatin (GelMA) meniscal scaffold using projection stereolithography that mimicked native collagen alignment [91]. The authors chose human avascular-zone meniscal cells to seed into the scaffolds for 2 weeks with a chondrogenic culture and then implanted the scaffolds into defective menisci. The 3-week postoperative results confirmed that the GelMA scaffolds were nontoxic and directed cell-aligned growth. Sarem et al. fabricated macroporous multilayered gelatin (G)/chitosan (Cs) scaffolds [92, 93]. Cs in conjunction with G not only enhances the bioactivity of Cs but also improves water retention and oxygen and nutrient transfer because of the hydrophilicity of G [94]. Ishida et al. investigated platelet-rich plasma (PRP) combined with gelatin hydrogel scaffolds to enhance meniscal regeneration [95]. PRP can be prepared easily from a patient's blood by centrifugation and is a rich source of growth factors, such as platelet-derived growth factor, insulin-like growth factor-1, and transforming growth factor *β*-I (TGF *β*-I) [96]. The findings showed that PRP enhances regeneration of a defective avascular meniscus. Similarly, Simson et al. tested bone marrow (BM) and chondroitin sulfate (CS) to improve the regenerative capacity of hydrogel scaffolds [97]. Their results showed that BM improves fibrochondrocyte viability, proliferation, and migration, whereas CS enhances adhesive strength and matrix production. In another study, Ballyns et al. constructed an anatomically shaped meniscus using alginate and investigated the interaction between media-mixed and engineered tissue [98]. The results confirmed that adequate mixing improves biomechanical properties and the accumulation of matrix in the engineered constructs.

## 6. Decellularized Meniscal Scaffolds

Decellularized meniscal scaffolds not only provide a suitable microenvironment for cells but also preserve appropriate meniscal geometry. However, some challenges should be addressed to obtain ideal meniscal scaffolds.

It is difficult for seed cells to evenly penetrate a decellularized meniscus. A high concentration of bone morphogenetic protein-2 (BMP-2), a member of the TGF-*β* superfamily, stimulates MSC differentiation and can affect cell migration. Minehara et al. used recombinant human bone morphogenetic protein-2 (rhBMP-2) loading in solvent-preserved human menisci to induce migration of chondrocytes into decellularized menisci [99]. The results showed that rhBMP-2 induces migration of chondrocytes and improves proteoglycan production* in vitro*. The seed cell distribution challenge could be addressed* in vitro* by taking full advantage of these kinds of exogenous chemokines.

Sandmann et al. used sodium dodecyl sulfate (SDS) as the main ingredient to decellularize human menisci [100], and the results showed that this method retains collagen structure. The results of a biomechanical assessment using a repetitive ball indentation test (stiffness, N/mm; residual force, N; relative compression force, N) on the processed tissue were similar to those of the intact meniscus, and the histological results showed no residual cells. Stapleton et al. attempted a complicated decellularized procedure consisting of freeze-thaw cycles, SDS, and disinfection using peracetic acid [101] to obtain decellularized scaffolds. As a result, the scaffolds demonstrated well-preserved structural proteins and biomechanical properties and were not cytotoxic.

Maier et al. used a self-developed enzymatic process to treat ovine menisci [102]. Their results suggested that native cells and immunogenic proteins (MHC-1/MHC-2) are completely removed while retaining significant biomechanical properties. Stabile et al. applied concomitant decellularization and oxidation processes to improve porosity [103]. Porosity of the decellularized scaffolds increased to some extent, and the scaffolds were not cytotoxic. In addition, Azhim et al. used a neoteric sonication decellularization system to produce decellularized bovine meniscal scaffolds [104]. These scaffolds had good biomechanical properties, similar to those of native meniscus, and the immunogenic cell components were removed. Nevertheless, the sonication treatment significantly changed the native ECM components and collagen fiber arrangement.

## 7. Future Prospects for Meniscal Regeneration

Future tissue-engineered meniscus strategies should focus on constructing an entire functional unit to maintain knee joint homoeostasis. Constructing an inferior biomechanical meniscus prevents proper knee joint function. Messner and Gao used menisci and their insertions into bone (entheses) to represent a functional unit [20], containing a meniscal body with anterior and posterior ligaments and entheses (Figure 1(b)). This unit may suggest the future development of a tissue-engineered meniscus.

Three-dimensional printing could benefit the development of ideal meniscal scaffolds with biomimetic structure and a beneficial microenvironment for cell growth. Lee et al. fabricated 3D-printed novel human meniscal scaffolds that recapitulated principal collagen alignment using PCL loaded with human connective tissue growth factor and TGF-*β*3 [105].

Meniscal anatomical requirements for orthopedic applications could be addressed by advances in imaging. Ballyns et al. generated a tissue-engineered meniscus for the first time based on meniscal anatomic geometry using microcomputed tomography and MRI [106]. Thus, a perfect combination of the meniscal unit, 3D printing, and medical imaging technology could direct future development of meniscal tissue engineering to achieve knee joint homoeostasis [107].

## 8. Conclusions

The study of scaffolds is the basis for meniscal tissue engineering. Nevertheless, a concerted effort must be made to explore other options, including seed cells and appropriate biological and biomechanical stimulation. It is insufficient to prepare scaffolds that are merely biomimetic to meniscal composition and structure. The key issue is how to obtain the excellent biomechanical function of the native meniscus. Future engineered menisci should combine these advantages to achieve an individualized tissue similar to that of the native meniscus.

## Figures and Tables

**Table 1:** Summary of main studies concerning the bioabsorbable synthetic polymer scaffolds.

Polymers type	Fabrication method	Cellular type	*In vitro* or *in vivo *	Stimulations	Time	Results	References
PGA bonded PLGA	Lyophilization	Allogeneic rabbit meniscal chondrocytes	*In vivo* (rabbit)	None	36 weeks	Proteoglycan types I and II collagen in neomenisci Differences in collagen content and aggregate modulus in comparison with native meniscus	Kang et al. (2006) [50]

Pu	Solvent leaching	None	*In vivo* (male Wistar rats back)	None	24 weeks	Unorganized collagen deposition in isotropic scaffolds Collagen alignment in anisotropic scaffolds	De Mulder et al. (2013) [51]

PLDLA/PCL-T	Solvent casting and particulate leaching	Rabbit meniscus fibrochondrocytes	*In vivo* (rabbit)	None	24 weeks	Without apparent rejection, infection, or chronic inflammatory response Mature collagen in seeded cells scaffolds	Esposito et al. (2013) [52]

PEO loaded collagenase	Electrospinning	None	*In vitro *	None	4 weeks	Improved repair by promoting cell migration, proliferation, and matrix deposition	Qu et al. (2013) [53]

PCL	Electrospinning	Juvenile bovine MSCs	*In vitro *	Collagenase and ChABC	120 days	Collagen dominated tensile response, GAG dominated compressive properties, and GAG removal result in significant stiffening in tension	Nerurkar et al. (2011) [54]

PCL mixed PEO	Electrospinning with rotating mandrel	Juvenile meniscus fibrochondrocytes	*In vitro *	Various porosity and preseeding	8 weeks	Highly porous scaffolds integrate better with a native tissue and mature, preseeding improved integration with the native tissue	Ionescu and Mauck (2013) [55]

HYAFF/PCL	Lamination technique	Autologous chondrocytes	*In vivo* (sheep)	Transosseous horns fixation	4 months	Better implant appearance was in without fixation group; significant cartilaginous tissue formation and lower joint degeneration was in cell-seeded group	Kon et al. (2008) [56]

HYAFF/PCL	Lamination technique	Autologous chondrocytes	*In vivo* (sheep)	None	12 months	Avascular cartilaginous formation was more frequent in cell-seeded constructs; OA was less in cell-seeded group than in meniscectomy group	Kon et al. (2012) [57]

Actifit	—	None	*In vivo* (sheep)	None	12 months	Promoting tissue ingrowth into porous scaffolds Friction coefficient of scaffolds decreasing to near native values	Galley et al. (2011) [58]

Actifit	—	None	Clinical cases (54 patients)	None	24 months	Significant improvements of pain and function scores; scaffold is safe and effective in treating lateral meniscus defects	Bouyarmane et al. (2014) [59]

Actifit	—	None	Clinical cases (18 patients)	None	24 months	Scaffold with chronic segmental medial meniscus deficiency is not only a safe procedure but leads to good clinical results	Schüttler et al. (2014) [60]

Actifit	—	None	Clinical cases (18 patients)	None	24 months	No deleterious effects on patients Inducing and promoting meniscal regeneration by normal chondrocytes and fibrochondrocytes Beneficial in decreasing the risk of progression to knee OA	Baynat et al. (2014) [61]

PLDLA/PCL-T: poly (L-co-D,L-lactic acid)/poly (caprolactone-triol).

PEO: poly (ethylene oxide).

HYAFF/PCL: hyaluronan-derived polymers obtained by a coupling reaction (Fidia Advanced Biopolymers, Abano Terme, Italy).

Actifit: acellular meniscal scaffold mainly composed of PU (20%) and PCL (80%) (Orteq Sports Medicine, London, UK).

**Table 2:** Summary of main studies concerning the absorbable scaffolds derived from biological components.

Biological components	Source	Cellular type	*In vitro* or *in vivo *	Stimulations	Time	Results	References
Collagen sponge	Purified porcine skins	Human meniscal cells	*In vitro *	TGF-*β*	14 days	Attached well and expanded with culture time Permissive for production of meniscus ECM and for growth factor responsiveness	Gruber et al. (2008) [68]

Collagen-GAG	Bovine tendon collagen type I and C6S	Human chondrocytes and meniscal cells	*In vitro *	PDGF-BB and TGF-*β*1	21 days	Increased contraction of collagen-GAG matrix induced by TGF-*β*1 Decrease in contraction resulting from PDGF-BB treatment	Zaleskas et al. (2001) [69]

Hyaluronan (HYAFF-11)	—	Bovine articular chondrocytes	*In vitro *	Rotary cell culture system	4 weeks	Outer stained for versican and type I collagen; inner positively stained for GAGs and types I and II collagen Outer stiffer in tension, inner stiffer in compression	Marsano et al. (2006) [70]

Collagen II/I, III	Porcine collagen	Ovine meniscal cells	*In vitro *	None	28 days	Cells attached well to both biomaterials and produced GAGs and collagen type I	Chiari et al. (2008) [71]

Hyaluronan (HYAFF-11)	—	Ovine meniscal cells	*In vitro *	None	28 days	Differences between the biomaterials were to cell distribution, morphology, and dynamics of GAGs synthesis	Chiari et al. (2008) [71]

C6S coated PLGA surfaces	Shark C6S	Human meniscus fibrochondrocytes	*In vitro *	Hypoxia	14 days	Fibrochondrocytes redifferentiation were enhanced by hypoxia independent of hypoxia inducible factor (HIF) and potentially involve the transcriptional activation of Sox-9	Tan et al. (2011) [72]

Collagen type I (CMI)	Achilles tendon of bovine	Human BMSC	*In vitro *	Biomechanical stimulation and perfusion	14 days	Cell proliferation can be enhanced using continuous perfusion Differentiation is fostered by mechanical stimulation	Petri et al. (2012) [73]

Collagen type I (CMI)	Achilles tendon of bovine	None	Clinical cases (25 patients)	None	10 years	Significant pain relief and functional improvement No development or progression of degenerative knee joint disease was observed in most cases	Monllau et al. (2011) [74]

Collagen type I (CMI)	Achilles tendon of bovine	None	Clinical cases (33 patients)	None	10 years	Pain, activity level, and radiological outcomes are significantly improved	Zaffagnini et al. (2011) [75]

Multilayered silk scaffolds	*Bombyx mori *silkworm cocoons	Human fibroblasts (outside) chondrocytes (inside)	*In vitro *	TGF-*β*3	28 days	Maintenance of chondrocytic phenotype with higher levels of GAGs and collagens Improved scaffold mechanical properties along with ECM alignment	Mandal et al. (2011) [76]

Multilayered silk scaffolds	*Bombyx mori *silkworm cocoons	Human bMSC	*In vitro *	TGF-*β*3	28 days	Higher levels of collagens and GAGs Enhanced biomechanics similar to native tissue	Mandal et al. (2011) [77]

Bacterial cellulose	*Gluconacetobacter xylinus *	3T6-swiss albino fibroblasts	*In vitro *	Compression bioreactor	28 days	Microchannels facilitated the alignment of cells and collagen fibers Collagen production was enhanced by mechanical stimulation	Martínez et al. (2012) [78]

Hyaluronan (HYAFF-11): (Fidia Advanced Biopolymers, Abano Terme, Italy).

Collagen II/I, III: Geistlich Biomaterials (Wolhusen, Switzerland). Two layers; the less porous layer consisted of collagen type I and type III; the porous layer consisted of collagen type II.

Collagen type I (CMI): distributed by Ivy Sports Medicine (ISM) (formerly known as ReGen Biologics).

## Abbreviations

AL: Aligned
BC: Bacterial cellulose
BM: Bone marrow
Cs: Chitosan
C6S: Chondroitin-6-sulfate
CS: Chondroitin sulfate
CTGF: Connective tissue growth factor
CMI: Collagen meniscus implant
DCE-MRI: Dynamic contrast-enhanced magnetic resonance imaging
ECM: Extracellular matrix
GAGs: Glycosaminoglycans
HIF: Hypoxia-inducible factor
IGF-I: Insulin-like growth factor
MHC 1/MHC 2: Major histocompability complex 1/Major histocompability complex 2
MRI: Magnetic resonance imaging
MSCs: Mesenchymal stem cells
GelMA: Methacrylated gelatin
*μ*CT: Microcomputed tomography
G: Gelatin
NA: Nonaligned
OA: Osteoarthritis
PDGF: Platelet-derived growth factor
PRP: Platelet-rich plasma
PET: Polyethylene terephthalate
PGA: Polyglycolic acid
PLA: Polylactic acid
PLGA: Polylactic co-glycolic acid
PCL: Poly (*ε*-caprolactone)
PU: Polyurethane
SDS: Sodium dodecyl sulfate
SIS: Small intestinal submucosa
TGF *β*-I: Transforming growth factor *β*-I.
